# Nanomaterials in diagnostics, imaging and delivery: Applications from COVID-19 to cancer

**DOI:** 10.1557/s43579-022-00257-7

**Published:** 2022-10-17

**Authors:** Neelkanth Bardhan

**Affiliations:** 1 The Koch Institute for Integrative Cancer Research, Massachusetts Institute of Technology, 500 Main St., Cambridge, 02142 MA USA; 2 Department of Biological Engineering, Massachusetts Institute of Technology, 77 Massachusetts Ave., Cambridge, 02139 MA USA

**Keywords:** COVID-19, Biomedical, Nanoscale, Nanostructure, Biological

## Abstract

**Abstract:**

In the past two decades, the emergence of nanomaterials for biomedical applications has shown tremendous promise for changing the paradigm of all aspects of disease management. Nanomaterials are particularly attractive for being a modularly tunable system; with the ability to add functionality for early diagnostics, drug delivery, therapy, treatment and monitoring of patient response. In this review, a survey of the landscape of different classes of nanomaterials being developed for applications in diagnostics and imaging, as well as for the delivery of prophylactic vaccines and therapeutics such as small molecules and biologic drugs is undertaken; with a particular focus on COVID-19 diagnostics and vaccination. Work involving bio-templated nanomaterials for high-resolution imaging applications for early cancer detection, as well as for optimal cancer treatment efficacy, is discussed. The main challenges which need to be overcome from the standpoint of effective delivery and mitigating toxicity concerns are investigated. Subsequently, a section is included with resources for researchers and practitioners in nanomedicine, to help tailor their designs and formulations from a clinical perspective. Finally, three key areas for researchers to focus on are highlighted; to accelerate the development and clinical translation of these nanomaterials, thereby unleashing the true potential of nanomedicine in healthcare.

**Graphical abstract:**

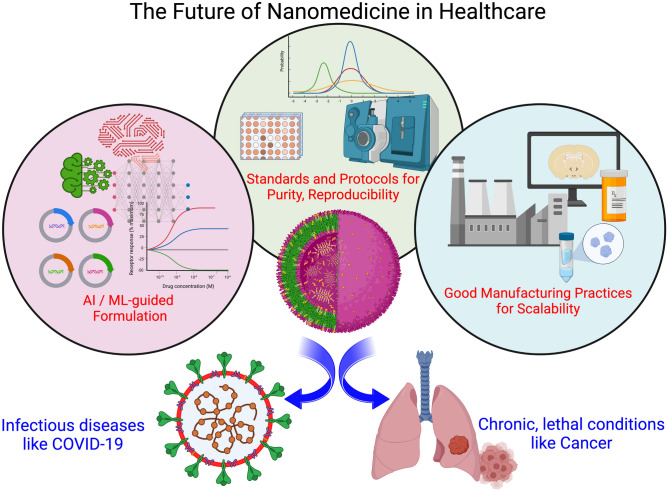

## Introduction

### What is nanotechnology?

“There’s plenty of room at the bottom”, stated Dr. Richard Feynman in his 1959 Caltech lecture at the American Physical Society annual meeting. In this remarkable talk, many of the ideas which are being actively studied and taken for granted today in the field of nanotechnology were hypothesized and put forth by Dr. Feynman: it should be possible, in principle, to arrange atoms the way we want by mechanical manipulation; tiny, ingestable surgical robots could be designed to “swallow the doctor”; scaling down letters to be small enough to fit the entire Encyclopedia Britannica on the head of a pin, and so on. However, the term “nano-technology” was first coined by Professor Taniguchi at the Tokyo University of Science,^[1]^ who defined the term as “nanotechnology mainly consists of the processing of separation, consolidation, and deformation of materials by one atom or one molecule. While Feynman and Taniguchi were among the first proponents of this new field, the term “nano” did not appear in the stream of public consciousness until the publication of the book “Engines of Creation: The Coming Era of Nanotechnology” by Dr. K. Eric Drexler, which proposed a nanoscale assembly technique whereby small molecular machines could build copies of themselves, and scale up to larger structures of arbitrary complexity, with precise control at the atomic scale.

**Figure 1:**
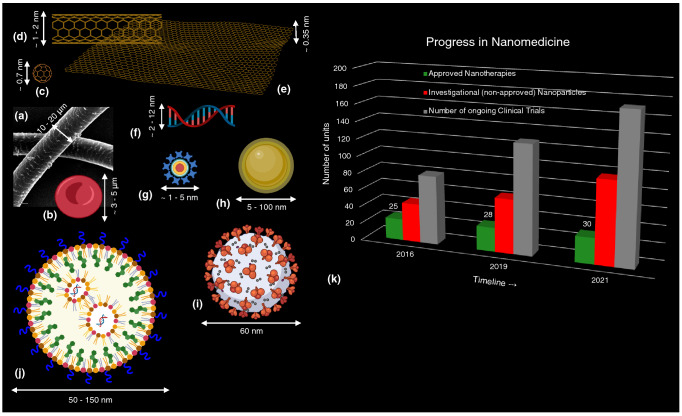
What is nanomedicine? A comparison of the different size scales, and a timeline of progress in the field. (a, b) Examples of structures in the human body: (a) a strand of human hair $\sim 10$–30 μm in diameter, (b) a red blood cell $\sim 3$–5 μm in size. Nanoscale materials range in size $\sim 1/10000\text {th}$–$1/100\text {th}$ of the thickness of a human hair. (c–e) Carbon-based nanomaterials: (c) 0D graphene dot $\sim 0.7$ nm, (d) 1D carbon nanotube $\sim 1$–2 nm in diameter, and (e) 2D sheet of graphene is $\sim 0.35$ nm in thickness. (f) A helix of double-stranded DNA is $\sim 2$–12 nm across. (g, h) Inorganic nanoparticles, such as: (g) quantum dots $\sim 1$–5 nm, while (h) gold nanoparticles $\sim 5$–100 nm in size. (i) The SARS-CoV-2 virus, responsible for the global COVID-19 pandemic, is $\sim 60$–140 nm in diameter. (j) In comparison, typical lipid nanoparticle formulations for the mRNA vaccines against COVID-19 are $\sim 50$–150 nm. Created with Biorender.com. (k) Accelerated rate of clinical progress in nanomedicine, since 2016. Data adapted from Anselmo and Mitragotri.^[3–5]^

On a more formal note, a generally accepted modern definition of nanotechnology, according to the National Nanotechnology Initiative, an agency of the US government, is as follows: “nanotechnology” may be defined as the control of matter at the nanoscale, with dimensions in the range of $\sim 1$–100 nanometers (nm). One nanometer (1 nm, or $10^{-9}$ m) is one-billionth of a meter. Just how small is a nanometer? Figure 1(a–j) shows the sizes of some common biomaterials for reference, and a few of the nanomaterials which are being widely studied and deployed in medical applications. A strand of human hair is of the order of 10–30 μm in diameter, while a human red blood cell is 3–5 μm in size. To qualify as a “nano” material, at least one dimension of the material needs to be in the range of $\sim 1/10,000$-1/100th the thickness of a strand of human hair. Some common examples of nanomaterials are: carbon-based nanomaterials, such as 0-dimension graphene dot (0.7 nm), 1-dimensional carbon nanotubes (1–2 nm in diameter), and 2-dimensional graphene sheet ($\sim 0.35$ nm in thickness). It is worthwhile to note that many biological molecules, designed by nature, also happen to be at the nanoscale level: the structure of DNA, for example, is a double-stranded helix with the distance between the strands ranging from 2 to 12 nm. Commonly used inorganic nanoparticles, such as gold nanospheres, range from $\sim 5$–15 nm, while fluorescent quantum dots are smaller at $\sim 1$–5 nm in size. Pathogens, too, can be nanoscale: for example, the SARS-CoV-2 virus was shown to be $\sim 60$–140 nm in size.^[2]^ So, too, are the vaccines developed to combat the COVID-19 pandemic: the lipid nanoparticle formulations of the mRNA vaccine are in the size range of $\sim 50$–100 nm, as depicted in Fig. 1(j).

### Why use nanotechnology in biomedical applications? Motivation and scope

The appeal of nanomaterials; compared to “bulk” or macro-scale materials, is that matter begins to exhibit unusual physical, chemical, electronic, optical or biological properties at the nanoscale; which is made possible by the structure and arrangement of atoms and molecules in this range of sizes and dimensions. For example, although bulk gold is a noble metal, and non-reactive with almost all chemicals except for very strong acid mixtures such as aqua regia, nanoscale gold can be highly reactive, and can be used to absorb, transfer and convert light energy into heat, with useful pharmacologic applications. In fact, the use of nanomaterials in medicine isn’t a new phenomenon: there have been various instances of the use of nanomaterials in ancient civilizations. The Lycurgus Cup, for example, dating back to the 4th century CE, gets its unique color patterns from the presence of colloidal Ag-Au nanoparticles which are $\sim 70$ nm in size dispersed in the glass. In the Medieval era, stained glass windows in churches and cathedrals used various nanoparticles to obtain a range of brilliant colors. More specifically, in the domain of medicinal uses, Au colloids have been long used and reported by scientists in China (used by physicians and surgeons to fight infections, fever, and measles^[6]^), India (Ayurveda Medicine^[7]^: the “Swarna Bhasma” ie. gold ash), and the middle east, as early as the 5th century BC. In fact, it has been proposed that Au nanoparticles have ushered in a “golden age” of biomedical nanotechnology^[8]^; one that is much more diverse in terms of the menu of available nanoparticles than the metallic nanostructures used in historical times.

In addition to their “nano” size (defined to be in the range of $\sim 1$–100 nm, as discussed in section “What is nanotechnology?”), biomedical nanotechnology offers some important benefits over their “bulk” or “macro-scale” counterparts: (a) The ability to tune the surface functionalization of these nanoparticles owing to the high surface area : volume ratio compared to bulk or macro-scale materials, resulting in multi-functional properties; (b) The ease of synthesis and fabrication of these materials, using a top-down or bottom-up approach,^[9]^ with a vast choice of form factors such as quantum dots, nanowires, thin films, or other shapes to suit the desired application; (c) The possibility to leverage novel bio-nano interaction mechanisms^[10, 11]^ of nanoparticles with human cellular- and tissue-level structures owing to their size similarities (as illustrated in Fig. 1), which can be optimized for achieving more efficacious treatment outcomes (offering the means to penetrate difficult obstacles in the body, such as the blood brain barrier^[12]^); (d) The opportunity to engineer “smart” nanomaterials, also known as stimuli-responsive nanosensors, which respond to specific triggers in their local environment (such as low pH, reduced oxygenation in tumors) to generate a detectable signal for scalable, low-cost, early, precision diagnostics; and (e) The potential for enhanced delivery of payloads (such as vaccines, drugs, or other biologics) using nanoscale carriers through improved solubility, longer circulation half-life, improved stability in the body, better targeting capability to the site of interest, and controlled drug-release profiles.

By using nanomaterials, researchers are equipped with the tools to study and manipulate macromolecules in real-time, and possibly offer interventions at the earliest stages of disease onset. Wouldn’t it be wonderful if a swarm of “nano robots” can identify rogue cells in the body, which are about to undergo tumorigenesis, and zap them to prevent the disease from taking hold in the first place? Such ideas, which may have been considered science fiction only a few decades ago, are slowly starting to become reality^[13]^ thanks to the advances in nanomedicine.

### The “coming of age” of nanomedicine: lessons from COVID-19, prospects in cancer

It has long been promised^[14]^ that the emergence of platforms based on nanomaterials can lead to the development and clinical translation of new classes of biological drugs (such as protein therapies, antibody drugs, checkpoint inhibitor immunotherapy, and most recently - gene editing through the CRISPR-Cas system), with the possibility of precise delivery at the site of disease, or even intracellular locations, for therapeutic efficacy. As depicted in Fig. 1(k), there are a great number of organic and inorganic nanomaterials under various stages of development^[3–5]^ with a wide range of applications in medicine.^[15]^ However, as the recent COVID-19 pandemic has demonstrated, some of the most spectacular successes in nanomaterial technologies have been made possible by polymeric nanoparticles, and liposomes, from a clinical standpoint. It is important to emphasize here that most nanomedicine formulations for clinical applications are not purely employed in their as-synthesized (nanoparticulate) form, but instead dispersed into a polymer or lipid matrix, forming a nanocomposite material with enhanced physico-chemical properties. Although there have been some clinically approved nanomedicines in recent years, such as Doxil (the liposomal formulation of the chemotherapy drug doxorubicin, which was the first nanomedicine approved^[16]^ by the U.S. FDA in 1995), it may be argued that the remarkable success achieved by the lipid nanoparticle formulations of the mRNA vaccines by Moderna Therapeutics and Pfizer-BioNTech, as well as the various nanomaterial-based platforms developed for rapid diagnostics of COVID-19 has led to the “coming of age” of nanomedicine,^[17]^ with their proven tolerability, safety and high efficacy profiles making them more acceptable in society, thereby paving the way for greater deployment of other types of nanotheranostics.

While there have been numerous reviews on the potential of nanotechnology in revolutionizing biomedicine^[18, 19]^ over the years, the sobering reality is that in many cases, nanomaterials have turned out to be more “hype” than reality.^[20]^ The present prospective study differs from the previous literature in three important ways: (1) With the benefit of hindsight, the advancements which led to the successful, rapid deployment of nanoscale assays for rapid diagnostic and testing platforms (section Nanomaterials for rapid diagnostics: point-of-care testing in COVID-19 and other infectious diseases, Table I), as well as lipid nanoparticle-based vaccines (section Lipid nanoparticles for vaccine delivery: success in COVID-19, progress in cancer, Table II) for COVID-19, are analyzed and highlighted here, which provides important learning value^[21]^ to nanomaterial researchers; (2) A compilation of the useful resources in the field of nanomaterials for biomedical applications is provided (Sect. Nanotechnology materials in medicine: useful resources for practitioners)—both for new practitioners to start thinking about the relevant considerations in nanomaterial formulations such as delivery (Section Delivery challenges), biocompatibility and toxicity (Sect. Mitigating toxicity concerns) with the goal of clinical translation while designing novel nanomaterials, and for established researchers to gain insights about already-existing products in the nanomedicine space and minimize duplication of effort; and (3) Key emerging areas of research are identified in a forward-looking agenda (Sect. Outlook and future prospects) using newly developed toolkits: such as the use of Machine Learning for combinatorial, high-throughput screening and predictive studies, the establishment of benchmarking standards and industry protocols to ensure purity and reproducibility, as well as the adherence to good manufacturing practices to scale-up production of nanomedicines; to accelerate the clinical translation of nanomedicines for cancer (Sect. Nanomaterials in cancer: diagnostics, therapy and treatment) and other disease conditions with the objective of benefiting patients.^[22]^

## Nanomaterials for rapid diagnostics: point-of-care testing in COVID-19 and other infectious diseases

The history of mankind has been marked with significant outbreaks of highly contagious, infectious diseases which have resulted in devastating pandemics, such as the Plague (Black Death, 1346-1353 AD), Spanish flu (H1N1 Influenza, 1918), smallpox, tuberculosis, HIV/AIDS (1980s—present), and most recently the ongoing COVID-19 (2020—present) outbreak caused by the SARS-CoV-2 virus. Given the rapidly transmissible nature of these (and other newly discovered, future potential) pathogens, there is a great unmet clinical need for the availability of low-cost, scalable testing platforms; both laboratory-based and point-of-care testing for mass screening at the population level. In this regard, nanomaterials have played a very significant role in helping us develop biosensors for rapid diagnostics; especially in the wake of the COVID-19 pandemic.^[23]^ A number of different detection techniques have been explored; such as those based on detecting a light signal (optical biosensors), changes in the heat signature (thermal biosensors), electrical properties of the medium (electrochemical biosensors), electron activity or redox potential (amperometric/potentiometric biosensors), or changes in surface potential (ion-sensitive biosensors), to name a few.

**Figure 2:**
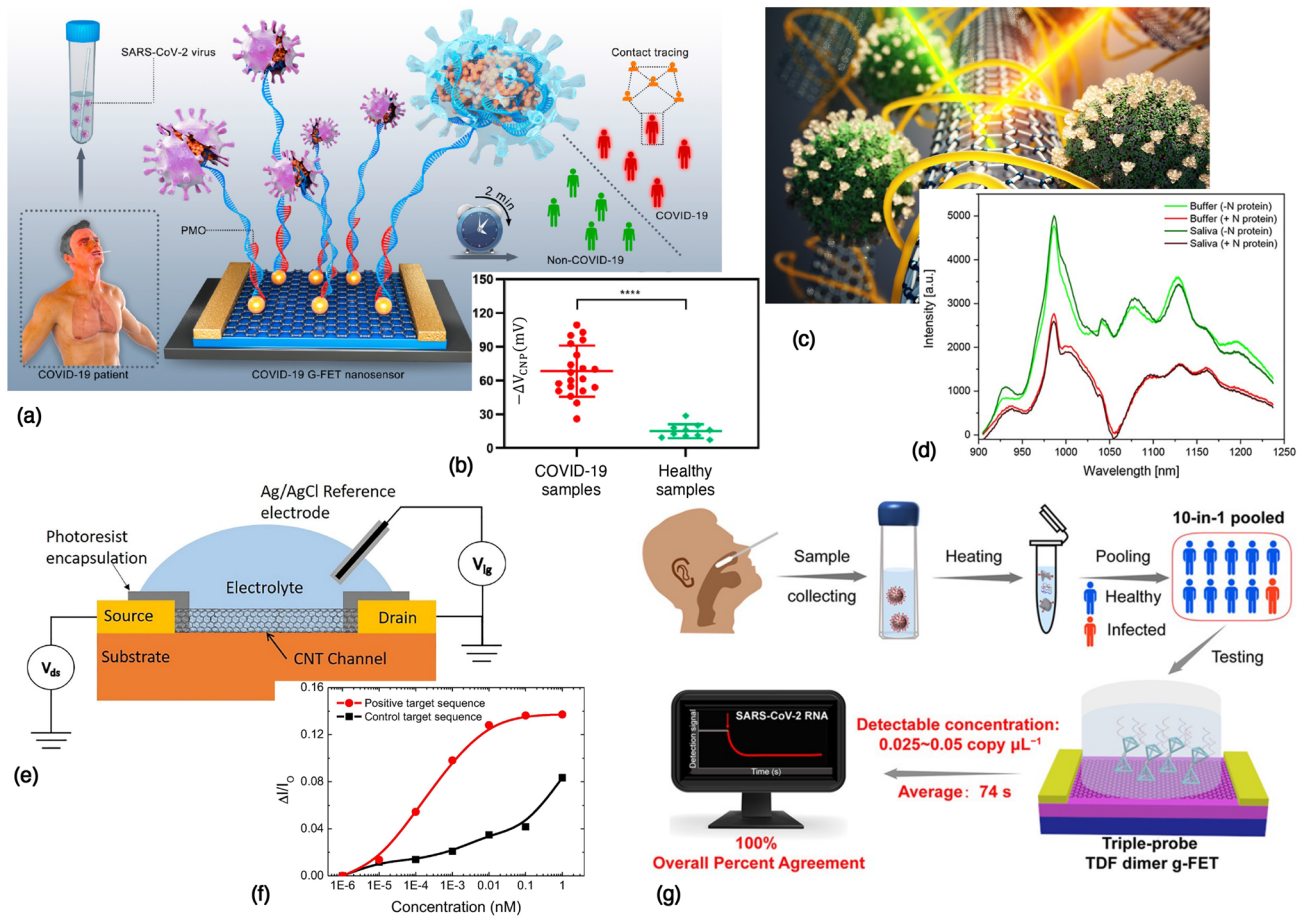
Working demonstrations of nanomaterials for rapid testing in COVID-19: (a) A PCR-free, rapid test ($< 2$ min) developed using PMO oligos on Au nanoparticle-decorated graphene field-effect transistor (g-FET), with a distinct signal response (b) in COVID-19 infected patients, compared to healthy individuals. Reprinted (adapted) with permission from Li et al.^[30]^ ©2021 Elsevier. (c) Antibody-free nanosensor, using PEG—phospholipid polymers adsorbed onto single-walled carbon nanotubes (CNTs). (d) The fluorescence spectrum of CNTs is used to identify the presence of the *N*-protein of the SARS-CoV-2 virus. Reprinted (adapted) from MIT News, and with permission from Cho et al.^[26]^ ©2021 American Chemical Society. (e) CNT-FET nanosensor, on a flexible printed circuit, for rapid detection of the SARS-CoV-2 RdRP gene, at a range of concentrations (f) down to 10 fM. Reprinted (adapted) with permission from Thanihaichelvan et al.^[27]^ CC BY-NC-ND 4.0. (g) A pooled, rapid testing ($\sim 74$s) framework for accurate, population-level screening of COVID-19 with high testing efficiency, using a triple-probe tetrahedral DNA framework on a g-FET. Reprinted (adapted) with permission from Wu et al.^[32]^ ©2022 American Chemical Society.

One class of nanomaterials which has generated a tremendous amount of excitement in the last few decades has been carbon-based nanomaterials,^[24]^ including fullerenes and carbon dots (0D), carbon nanotubes (1D), graphene and its derivatives (2D). In this section, we look at some of the examples of rapid diagnostic devices being developed for COVID-19, using different nanomaterial platforms; as listed in Table I. For example, carbon nanotubes have been used as a fluorescent signal to enable optical detection of the SARS-CoV-2 virus. In one instance, Pinals et al. have developed a protein-SWNT nanosensor,^[25]^ which can bind to the spike protein’s receptor-binding domain (RBD) on the SARS-CoV-2 virion. These nanosensors can provide a readout in $\sim 90$ min, with a lower limit of detection of 12.6 nM of the spike protein. In another instance, Strano et al. have developed a technique called Corona Phase Molecular Recognition (CoPhMoRe), which leverages a layer of amphiphilic polymer bound on a carbon nanotube, forming a looped layer called the “corona”; depicted in Fig. 2(c). Upon binding of the nucleocapside (*N*) or spike (*S*) proteins of the SARS-CoV-2 virus, the fluorescence properties of the underlying SWNT change, which results in an optical readout,^[26]^ as shown in Fig. 2(d). Using this approach, they were able to detect concentrations as low as $\sim 2.4$ pg of protein per ml, with a very rapid diagnostic test ($< 5$ min). Another example of using carbon nanotubes for detection^[27]^ of SARS-CoV-2 was reported by Thanihaichelvan et al. based on a CNT field-effect transistor as shown in Fig. 2(e, f). The CNTs are fabricated on a polymer substrate, and by immobilizing the complementary sequence of the RdRp gene on the CNT channel, a transistor with a concentration-dependent gating response is achieved. While the authors were able to demonstrate a lower limit of detection of $\sim 10$ fM using this electrochemical nanosensor, the performance of the sensor on human clinical samples remains to be validated, in comparison to standard assays such as the RT-PCR diagnostic. For a comprehensive overview of the various advantages and limitations of the common biosensors based on carbon nanotubes currently being employed for detection of SARS-CoV-2, the reader is referred to the recent review by Varghese et al.^[28]^

**Table I:** Selected nanomaterial-based platforms for rapid diagnostics in COVID-19.

Disease condition	Analyte to be detected	Nanosensor material	Principle of detection	Required sample	Detection time	Limit of detection	Working range	Sensitivity/specificity
COVID-19	RdRp, spike protein	AuNTs^[29]^	Electrochemical	Nasopharyngeal swab	Unknown	22.2 fM	Not specified	Not specified
COVID-19	RdRp gene	g-FET^[30]^	Transfer curve shift	Throat swab/serum	$\sim 1$ h	2.29 fM (throat), 3.99 fM (serum)	10 fM–10 pM	$> 0.97$/1.00
COVID-19	S protein	Peptide on SPAuE^[31]^	EIS	Nasopharyngeal swab	$\sim 15$ min	18.2 ng/ml, 0.01 copies/ml	0.05–1.0 μg/ml, $10^2$–$10^3$ copies/ml	Not specified
COVID-19	SARS-CoV-2 RNA	g-FET^[32]^	Transistor response (I/V changes)	Nasopharyngeal swab	1–4 min	0.025-0.05 copy/μl	Not specified	0.93/1.00
COVID-19	SARS-CoV-2 Orf1ab, N genes	AIEgen@GO^[33]^	Fluorescence enhancement	Not specified	$\sim 1$ h	200/100 pM for Orf1ab/N cDNA	Not specified	Not reported
COVID-19	SARS-CoV-2 RNA	rGO^[34]^	Fluorescence	Pharyngeal swab	$\sim 35$ min	0.684 pM	1 pM–25 nM	Not reported
COVID-19	N, S protein	CNT^[26]^	nIR Fluorescence	Saliva	$\sim 5$ min	48 fM of N, 350 pM of S protein	0.1–100 nM	Not reported
COVID-19	Spike protein RBD	CNT^[25]^	Fluorescence	Not specified	$\sim 90$ min	12.6 nM of spike RBD	1 nM–1 μM	Not reported
COVID-19	RdRp gene	CNT-FET^[27]^	Current response	Not specified	$\sim 10$–30 min	10 fM	10 pM–1 nM	Not reported
COVID-19	*N*-gene	g-ssDNA-AuNP^[35]^	Voltage change	Nasopharyngeal swab	$< 5$ min	6.9 copies/μl of viral RNA	585.4-$5.854 \times 10^7$ copies/μl	$100\%$/$100\%$
COVID-19	Viral oligonucleotides	AuNIs^[36]^	LSPR, plasmonic photothermal heating	Not specified	$\sim 30$ min	$0.22 \pm 0.08$ pM	0.1 pM–1 μM	Not specified
COVID-19	IgM and IgG antibodies	Au^[37]^	Colorimeteric	Whole blood, serum or plasma	$\sim 15$ min	Not reported	Not known	$88.66\%$/$90.63\%$

*SARS-CoV-2* severe acute respiratory syndrome coronavirus-2, *RdRp* RNA-dependent RNA polymerase, *RBD* receptor binding domain, *CNT* carbon nanotubes, *nIR* near infrared, *GO* graphene oxide, *rGO* reduced graphene oxide, *SPAuE* screen-printed gold electrode, *AuNTs* gold nanotriangles, *AuNIs* gold nanoislands, *LSPR* localized surface plasmon resonance.

AS briefly discussed in Sect. 1.2, one of the well-explored classes of nanomaterials is Au nanoparticles, and its various derivatives; because of the ease of chemical functionalization for detection of a wide range of analytes, and the ability to generate an optical signal based on the phenomenon of plasmon resonance. For example, del Caño et al. have reported an electrochemical sensor^[29]^ based on gold nanotriangles (AuNTs), decorated with a di-thio DNA capture probe, for binding and detection of the RdRp and spike proteins of the SARS-CoV-2 virus. This amplification-free detection method was reported to have a lower limit of detection of $\sim 22.2$ fM. Recently, Soto and Orozco have reported an “label-free” electrochemical biosensor based on a synthetic peptide on a screen printed Au electrode.^[31]^ Using electrochemical impedance spectroscopy, the authors were able to detect the spike protein of SARS-CoV-2 in $\sim 15$ min., down to 0.01 copies/ml. Another unique label-free approach, deployed by Qiu et al. capitalized on the combined detection capability of localized surface plasmon resonance (LSPR) signal with the heat generation capability of plasmon-induced photothermal effect,^[36]^ for detection of the SARS-CoV-2 virus, with a lower limit of detection down to 0.22 pM. The use of the plasmonic photothermal heating allowed the researchers to improve the specificity of hybridization of the virus’ RdRp sequence with the complementary DNA, which enables the nanosensor to distinguish SARS-CoV-2 from related coronaviruses such as SARS-CoV. While many of the aforementioned assays have worked on detecting specific sequences or parts of the viral protein, antibody-based diagnostic assays have also gained considerable popularity in recent times for their rapid turnaround. In one of the earliest reports of an antibody diagnostic assay for the SARS-CoV-2 pathogen, Li et al. fabricated an lateral flow immunoassay chip^[37]^ with Au nanoparticles conjugated with SARS-CoV-2 recombinant protein, which offered a fast, rapid point-of-care diagnostic for detecting both IgM/IgG antibodies against the virus in serum from whole blood. While requiring only about $20 \upmu$l of whole blood sample, the assay has a readout within $< 15$ min. based on a visual color change, and very good performance with $\sim 89\%$ sensitivity and $\sim 91\%$ specificity.

Another exciting class of rapid diagnostics which has gained considerable traction in recent years has been the graphene-based platforms; which include graphene and its derivatives, namely graphene oxide (GO) and reduced graphene oxide (rGO). For an excellent discussion on the use of graphene-based nanosensors in various aspects of human health monitoring, the reader is referred to the comprehensive review by Huang et al.^[38]^ In one instance, Li et al. have designed amplification-free rGO-based field-effect transistor nanosensors,^[30]^ using Au nanoparticles decorated on the rGO, functionalized with complementary phosphorodiamidate morpholino oligos (PMO) as depicted in Fig. 2(a). This sensor works on the principle of hybridization of the PMO with the SARS-CoV-2 RdRp gene, which can be detected rapidly ($< 2$ min.). With a lower limit of detection of $\sim 2.29$ fM in throat swab samples and $\sim 3.99$ fM in serum, this sensor is highly sensitive, specific, and as shown in Fig. 2(b), shows outstanding accuracy in distinguishing COVID-19 from healthy samples when compared with the benchmark RT-PCR assay; which makes this a very promising candidate for a rapid, highly accurate test for health screening in point-of-care settings. In another example, Wu et al. have developed^[32]^ a graphene field-effect transistor (g-FET), functionalized with a triple-probe tetrahedral DNA framework (TDF), for 10-in-1 pooled testing of SARS-CoV-2 RNA as illustrated in Figure 2(d). Upon testing on clinical samples of nasopharyngeal swabs, the researchers were able to detect as few as 0.025–0.05 copies of viral RNA/$\mu$l, which is lower than the detection limit of RT-PCR, with a very fast readout in $\sim 1$–4 min., and a high sensitivity of 0.93 and perfect specificity. This method of using un-amplified, pooled testing based on graphene nanosensors has the great potential for rapid scale-up of a high-throughput screening for large populations, with applications in COVID-19 as well as in future pandemics. In another example, Zhang et al. have synthesized a nanosensor^[33]^ based on aggregation-induced emission luminogens (AIEgen)-labeled oligonucleotides, on GO substrates, for a 1-step detection method to identify the *ORF1ab*- or *N*-genes of the SARS-CoV-2 virus. Based on the fluorescence signal enhancement, the authors were able to detect signal down to 200 or 100 pM respectively for the two genes, with good sensitivity, in a relatively short time of $\sim 1$ h, which shows promise for use as a point-of-care testing. Another example of a rapid, ultra-sensitive detection platform^[35]^ was reported by Alafeef et al. based on a graphene sensor decorated with Au nanoparticles and coated with antisense oligonucleotides specific to the *N*-gene, relying on surface-enhanced Raman scattering to detect the SARS-CoV-2 virus. In $< 5$ min, this diagnostic assay was able to achieve detection down to 6.9 copies/$\upmu$l; with $100\%$ sensitivity, specificity and accuracy without the need for amplification. Another example we highlight in Table I is a fluorescence assay developed by Wang et al. based on rGO,^[34]^ with rapid detection capability in $< 35$ min, without the need for RNA amplification, allowing a lower limit-of-detection of $\sim 0.684$ pM. More work needs to be done to improve the sensitivity of these easy-to-fabricate, high-throughput nanosensors for detection of biological analytes, with the goal of achieved continuous monitoring and testing at the whole population level, in order to successfully manage pandemics such as COVID-19.

## Lipid nanoparticles for vaccine delivery: success in COVID-19, progress in cancer

It is generally well-accepted among the public health community that vaccination is considered to be the most effective tool for combating disease-associated mortality and morbidity. In the race to control and mitigate the effects of the COVID-19 pandemic affecting the whole world, messenger ribonucleic acid (mRNA) vaccines delivered through a formulation encapsulated in lipid nanoparticles (LNPs) have emerged as a new star,^[39]^ with outstanding $\sim 95\%$ efficacy reported by both the Pfizer-BioNTech BNT162b2 vaccine^[40]^ and the Moderna mRNA-1273 SARS-CoV-2 vaccine^[41]^; heralding the dawn of the next-generation of biologic drugs made possible by advances in this nanoparticle technology. In fact, it is quite remarkable to note the pace at which the rollout of the COVID-19 vaccine has surpassed the $50\%$ coverage^[42]^ of the global population within about a year of introduction; in comparison, other vaccines for measles, diptheria, pertussis, etc. took nearly 10–20 years to achieve the same level of penetration. The use of polymers for creating nanoparticles for the sustained release of proteins and other large biological macromolecules is certainly not new^[43]^; and has been explored for the last 4 decades.^[44]^ Why, then, is there such great excitement in the field of nanomedicine regarding the advent of lipid nanoparticle formulations, and what makes them special^[45]^? The secret lies in the ease of formulation, and the plug-and-play nature of LNP nanoparticles: it is regarded as a “platform technology”, in the sense that LNP-mRNA vaccines can be manufactured against specific diseases in a very short period of time, which makes them uniquely suited to respond to emerging infectious diseases caused by pathogens such as SARS-CoV-2.

**Figure 3:**
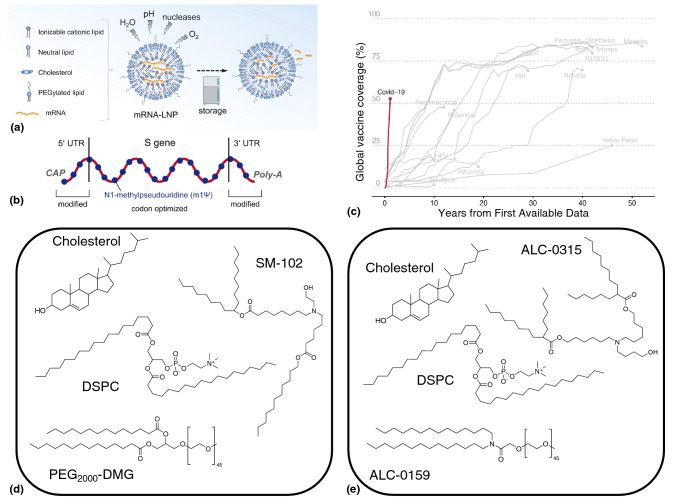
Fine tuning the nanomaterial formulations for successful delivery of mRNA-based COVID-19 vaccines: (a) Lipid nanoparticles protect the mRNA from environmental degradation factors, and during storage. Reproduced (adapted) from: Schoenmaker et al.^[46]^ CC BY 4.0. (b) The modified structure of the mRNA in both the Pfizer-BioNTech and Moderna vaccines, with all uridines replaced by N1-methylpseudouridine (m1$\Psi$) to reduce innate immune responses and increase its stability. Reproduced (adapted) from: Heinz and Stiasny^[47]^ CC BY 4.0. (c) The fastest global large-scale rollout of the COVID-19 vaccines; relative to all other vaccines in history. Reproduced (adapted) from: Glassman et al.^[42]^ CC BY-NC 4.0. (d, e) Structures of the lipids used in the LNP formulations of the (d) Moderna and (e) Pfizer-BioNTech COVID-19 vaccines.

So how do lipid nanoparticles enable the delivery of the COVID-19 vaccines? These vaccines comprise in vitro-transcribed mRNA strands^[48]^ encapsulated in LNPs, mainly to improve their stability, since naked mRNA delivered in the body is quickly degraded before they can be taken up by the host cells for antigen expression. As shown in Fig. 3(c, d), the lipids used in the formulation of these LNPs typically comprise : an ionizable cationic lipid, a PEGylated lipid, cholesterol, and the phospholipid DSPC as the helper lipid. The cholesterol component can increase the particle stability by imparting rigidity to the membrane, thereby ensuring integrity of the LNP. The PEG-lipid component, such as PEG$_{2000}$-DMG (1,2-dimyristoyl-rac-glycero-3-methoxypolyethylene glycol-2000) or ALC-0159 (2-[(polyethylene glycol)-2000]-*N*,*N*-ditetradecylacetamide) is used to control the final particle size of the LNP, and act as a steric barrier to mitigate aggregation of these particles during storage of the vaccine. The cationic lipid component, such as SM-102 (heptadecan-9-yl 8-((2-hydroxyethyl)(6-oxo-6- (undecyloxy)hexyl)amino) octanoate) and ALC-0315 (((4-hydroxybutyl)azanediyl)*bis*(hexane-6,1-diyl)*bis*(2-hexyldecanoate)) in the Moderna and Pfizer vaccines, respectively, consists of positively charged ionizable amine groups which are protonated at low pH, but remain neutral at physiological pH. This feature is beneficial for the delivery of mRNA into the cells, because the neutral lipids have less interactions with the anionic membranes of blood cells, and are more biocompatible. However, once inside the endosome inside a host cell, the pH is more acidic, which causes the ionizable lipid to become positively charged. This destabilizes the membrane, and faciliates endosomal escape of the nanoparticles,^[49]^ with the dumping of the cargo (mRNA) into the cytoplasm for subsequent translation into the target protein of interest.

Building upon the success achieved in delivering mRNA vaccines for COVID-19, companies such as Moderna Tx and BioNTech have a robust pipeline of new mRNA drugs in various phases of development, against a wide range of conditions; including respiratory vaccines, cancer vaccines, intra-tumoral immunotherapy, secreted and cell surface therapeutics, systemic therapeutics, as well as treatments against infectious diseases such as HIV, malaria, tuberculosis, and influenza, to name a few. Table II shows a summary of the various LNP-based formulations of mRNA biologics as vaccines in infectious diseases and cancer.

**Table II:** Lipid nanoparticle-based formulations of selected RNA therapeutics currently under clinical investigation.

Disease condition	Formulation and mRNA dose (when known)	Company/entity	Clinical trial phase	Clinical trial registry
SARS-CoV-2	BNT162b2 (30 $\upmu$g)	Pfizer/BioNTech SE	EUA/CMA	NCT04368728
SARS-CoV-2	mRNA-1273 (100 $\upmu$g)	ModernaTX, Inc., BARDA, NIAID	EUA/CMA	NCT04470427
SARS-CoV-2	CVnCoV (12 $\upmu$g)	CureVac AG	III	NCT04652102
SARS-CoV-2	ARCT-021 (7.5 $\upmu$g or 2$\times$ 5 $\upmu$g)	Arcturus Therapeutics, Inc.	II	NCT04728347
SARS-CoV-2	DS-5670a (10, 30, 60 or 100 $\upmu$g)	Daiichi Sankyo Co., Ltd.	I/II	NCT04821674
SARS-CoV-2	LNP-nCoVsaRNA (2.5, 5 or 10 $\upmu$g)	Imperial College London	I	ISRCTN17072692/2020-001209-22
SARS-CoV-2	MRT5500 (up to 13.5 $\upmu$g)	TranslateBio, Sanofi	I/II (abandoned)	NCT04798027
Zika virus	mRNA-1893 (30 or 100 $\upmu$g)	ModernaTX, Inc.	II	NCT04917861
Chikungunya virus	VAL-181388/mRNA-1388 (25, 50 or 100 $\upmu$g)	ModernaTX, Inc., DARPA	I	NCT03325075
H10N8 (Influenza)	VAL-506440/mRNA-1440 (25 or 100 $\upmu$g)	ModernaTX, Inc.	I	NCT03076385
H7N9 (Influenza)	VAL-339851/mRNA-1851 (10, 25 or 50 $\upmu$g)	ModernaTX, Inc.	I	NCT03345043
Rabies	CV7202 (2$\times$ 1 $\upmu$g)	CureVac AG	I	NCT03713086
Heart failure	AZD8601 (3 mg or 30 mg)	AstraZeneca	II	NCT03370887
Transthyretin-related (ATTR) familial amyloid polyneuropathy, cardiomyopathy, wild-type TTR cardiac amyloidosis	NTLA-2001 (0.1, 0.3, 0.7 or 1.0 mg/kg)	Intellia Therapeutics	I	NCT04601051
TTR-mediated amyloidosis, amyloidosis, hereditary, transthyretin-related	ALN-TTR02 (siRNA dose: 0.3 mg/kg, every 3 weeks for 18 mo.)	Alnylam Pharmaceuticals	FDA-approved (Onpattro/patisiran)	NCT01960348
Melanoma, colon, gastrointestinal, genitourinary or hepatocellular cancer	NCI-4650 (40, 130 or 390 $\upmu$g)	National Cancer Institute (NCI)	I/II	NCT03480152
Melanoma	FixVac (BNT111) (14.4, 50 or 100 $\upmu$g)	BioNTech SE	I	NCT02410733
Non-small-cell lung cancer (NSCLC)	mRNA-5671/V941 (dose unknown)	Merck Sharp & Dohme Corp.	I	NCT03948763
Triple-negative breast cancer (TNBC)	IVAC_W_bre1_ulD or IVAC_M_ulD (dose unknown)	BioNTech SE, Seventh Framework Program	I/II	NCT02316457
HPV-positive cancers (squamous cell carcinoma, head & neck neoplasm, cervical neoplasm, etc.)	HARE-40 (dose unknown)	University of Southampton, BioNTech SE	I/II	NCT03418480
Ovarian cancer	W_ova1 Vaccine (50 $\upmu$g, followed by 100 $\upmu$g total)	University Medical Center Groningen, BioNTech SE	I	NCT04163094
Solid tumors, cancer	MEDI1191 (dose unknown)	MedImmune LLC	I	NCT03946800

*SARS-CoV-2* severe acute respiratory syndrome coronavirus-2, *FDA* United States Food and Drug Administration, **EUA** emergency use authorization, *CMA* conditional marketing authorization.

## Nanomaterials in cancer: diagnostics, therapy and treatment

In this section, a brief overview of the work done in the author’s research group, towards the evolution of nanoscale materials as probes for cancer theranostics, is provided. Early detection of cancer is of utmost importance, as it is estimated that $\sim 50\%$ of cancers are detected at an advanced stage^[50]^; when treatment becomes more challenging. One of the areas of focus in recent years, towards the development of nanoparticles suitable for diagnostic and therapeutic applications, is the development of bio-functionalized nanoparticles, owing to their unique advantages such as: (a) active targeting, for highly specific detection of the analyte or disease of interest; (b) modular tunability, for targeting a wide range of pathogens, cancers, or other abnormalities; and (c) the ability to add functional groups on the surface of the nanoparticles without the need for chemical modifications, which makes them more bio-compatible and suitable for in vivo duties.

### Nanomaterials for cancer imaging and diagnostics

**Figure 4:**
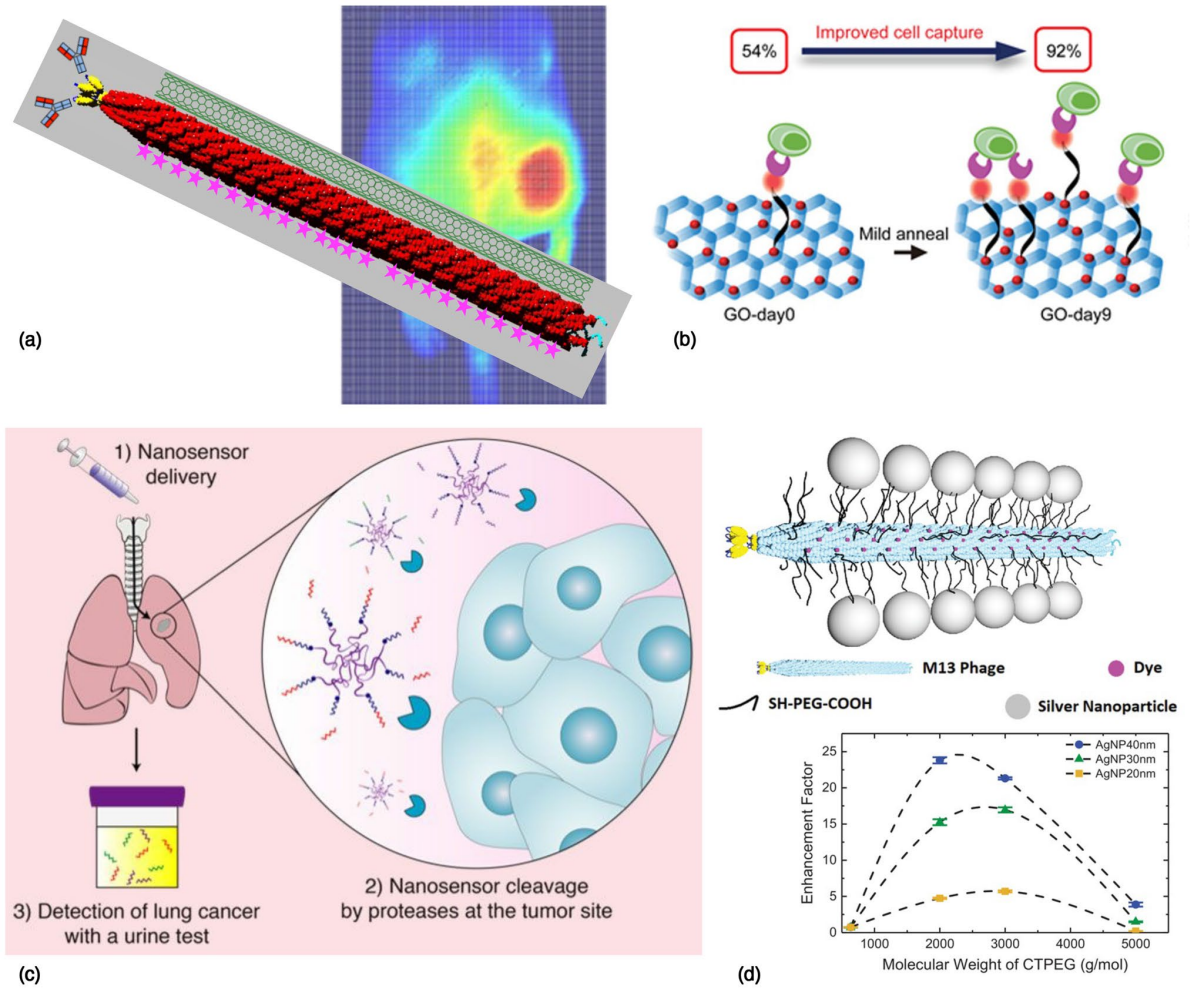
Designing nanomaterials for cancer diagnostics: (a) A “bio-templated” nanoprobe, M13 bacteriophage (in red) used to deliver a payload of interest such as dye molecules (purple), or a carbon nanotube (green), using antibody targeting. Shown here is an implementation to image an intramuscular mouse model of bacterial infection (glowing red spot on the right flank). Reprinted (adapted) from Bardhan et al.^[52]^ (b) A microfluidic-free, planar device created on treated graphene oxide (GO) nanosheet substrates, for quick and efficient capture of cells from whole blood; with enhanced capture efficiency from $\sim 54\%$ to $\sim 92\%$ using a mild thermal annealing process. Reprinted (adapted) from Bardhan et al.^[55]^ ©2017 American Chemical Society. (c) A “smart” activity-based nanosensor, developed as a non-invasive urine-based diagnostic for lung cancer. Upon delivery to the lungs, the reporter molecules get cleaved by tumor-associated proteases and excreted in the urine. Reprinted from MIT News, content from Kirkpatrick et al.^[56]^ ©2020 The American Association for the Advancement of Science. (d) Example of an application of a nanotemplate for plasmonic fluorescence enhancement: with dye molecules and Ag nanoparticles decorated on M13 phage, up to $\sim 24\times$ enhancement can be achieved. Reprinted (adapted) with permission from Huang et al.^[53]^ ©2019 John Wiley and Sons.

In one example, Bardhan et al. have utilized engineered strains of the bacteriophage M13, as a biological scaffold capable of simultaneously functionalizing the nanoparticle of interest (such as Au nanoparticles, carbon nanotubes, iron oxide nanoparticles, or dye molecules, to name a few) as well as to deliver the payload through targeting motifs engineered via peptide display on the outer surface of the virus. Using this bio-template, they have demonstrated the ability to use M13-SWNT imaging probes for detection and non-invasive imaging of deep tissue bacterial infections,^[51]^ both in an intramuscular model of infection as shown in Fig. 4(a), and in a model of *Staphylococcus aureus* endocarditis.^[52]^ In another example, these bacteriophages have been used to improve the fluorescence of Cy3 dye by a factor of $\sim 24\times$, by decorating silver nanoparticles on the M13 backbone as depicted in Fig. 4(d), and varying the size and Ag particle-fluorophore separation to optimize the enhancement^[53]^ achieved by plasmonic resonance. Coupled with the bright emission of these nanomaterials, they have also developed next-generation imaging systems,^[54]^ which can identify fluorophores in deep tissue up to 8 cm, and can enable real-time non-invasive tracking of a 0.1 mm-sized fluorophore through the GI tract of a living mouse; which pushes the boundaries of optical imaging for diagnostic applications.

In another example, Bardhan et al. have exploited the ability to easily modify and add functional groups on the surface of two-dimensional nanomaterials such as graphene and its derivatives, graphene oxide (GO) or reduced graphene oxide (rGO), to develop nanotemplates for unique bio-focused applications such as cell capture from whole blood. Through a simple one-step process of thermal annealing,^[57]^ with no chemical treatments involved, they have shown that it is possible to redistribute the oxygen functional groups on the surface of GO through a phase transformation process, while preserving the total oxygen content, which makes it a very useful material for additional functionalization. This method can also be extended to rGO, with the ability to impact the number of carbon and oxygen atoms removed during the reduction process by tuning the degree of oxygen clustering prior to the reduction step.^[58]^ Based on this two-dimensional nanomaterial, researchers have developed a GO-based cell capture assay, by coating the surface with single-domain antibodies, or “nanobodies”, for efficient capture of white blood cells from small sample volumes ($\sim 30\upmu$l) of whole blood.^[59]^ Further, as illustrated in Fig. 4(b), they have shown that a microfluidic-free, planar device constructed on these “treated” GO nanosheets (enhanced by oxygen clustering using the mild thermal annealing treatment) can be used to improve the reactivity and the density of functionalization of the cell capture agents; allowing capture of Class-II MHC-positive cells from whole blood at an efficiency of $\sim 92 \pm 7\%$ without fractionation of the blood.^[55]^ These techniques provide compelling examples of the unique properties of nanomaterials which can be leveraged for improving the efficiency of bio-analytic assays, while providing a scalable, cost-effective approach to low-cost diagnostics.

Other groups of researchers have come up with unique ways to engineer “smart” nanomaterials for diagnostic applications—which respond to specific stimuli to generate a detectable signal. These stimuli could be endogenous, ie. present in the local microenvironment in the tumor or site of infection (such as low pH, reduced oxygen or hypoxia, to name a few), or exogenously applied, ie. externally stimulated (such as light-, heat-, ultrasound-, or magnetic field-activated) nanosensors. For example, work done by Bhatia, S.N. and co-workers has resulted in the development of a class of multiplexed activity-based nanosensors (ABNs), which when delivered via the intrapulmonary route,^[56]^ is capable of early detection of lung cancer with very high sensitivity ($\sim 95\%$) and specificity ($\sim 100\%$); working on the principle of protease-activated cleavage as illustrated in Fig. 4(c), offering a simple urine-based diagnostic readout. Building on this idea, Hao, L. and co-workers have developed a “PRISM” platform (protease-responsive imaging sensors for malignancy),^[60]^ which can combine the utility of ABNs carrying a synthetic biomarker with co-delivery of an imaging agent for PET-CT imaging or for urine-based readout of the signal, which can be used as a tool for precision diagnostics in a wide range of cancer types.

### Nanomaterials for drug delivery in cancer

Why are nanomaterials considered an attractive platform to achieve drug delivery in cancer and other diseases? There are several reasons: (a) Nanoparticles can lead to improved solubility of cargo, especially of large molecules (such as mRNA, siRNA, antibodies or other protein drugs) which may be hydrophobic; (b) Nanoparticles provide an outer layer of functionalization, which helps improve the stability and protects these molecules from enzymatic, pH or other environmental factors which may cause premature degradation prior to them reaching the target disease site in the body; (c) Nanoparticles can be decorated with additional functional groups or targeting moeities, which may promote in cell internalization, immune avoidance, membrane transport, increased residence or circulation times, or lower systemic toxicity among other functions, which could result in better tolerability, safety and efficacy of the treatment.

Generally speaking, for the purposes of classifying nanomedicines for drug delivery applications, there are 3 main classes: (i) Inorganic nanoparticles: these include molecules such as quantum dots, Au or Ag nanoparticles, graphene, GO, and its derivatives, iron oxide and its derivatives, mesoporous silica nanoparticles, and so on; (ii) Polymeric nanoparticles: such as polymer micelles, polymerosomes, dendrimers, nanospheres, etc.; and (iii) Lipid nanoparticles: including liposomes, emulsions, and the LNPs used for vaccine delivery, such as the ones discussed in Sect. 3. Each of these types of drug delivery nanocarriers offers significant advantages over the other. For example, inorganic nanoparticles offer the benefits of simple synthesis and physico-chemical functionalization techniques,^[61]^ excellent reproducibility, and good safety and biocompatibility profiles. Furthermore, these inorganic NPs can be tailored to be “smart” drug delivery systems,^[62]^ with drug delivery only in response to certain stimuli (such as pH, redox, enzymatic cleavage, or externally applied fields such as temperature, ultrasound, light, heat, or magnetic field). In comparison, polymer NPs offer the advantages of being highly biocompatible; offering the possibility of synthesizing naturally bio-degradable systems^[63]^ which are non-toxic, without eliciting a strong immune response, and can be cleared via enzymatic or other degradation mechanisms through metabolic pathways. Finally, lipid nanoparticles are an exciting class of nanotherapeutics in their own right; owing to the success in vaccine delivery in the context of COVID-19 as discussed in the previous section. LNPs offer the strongest advantages^[64]^ in their ease of scale-up for production, and the ability to deliver both hydrophilic and hydrophobic/lipophilic molecules with controlled release patterns.

**Figure 5:**
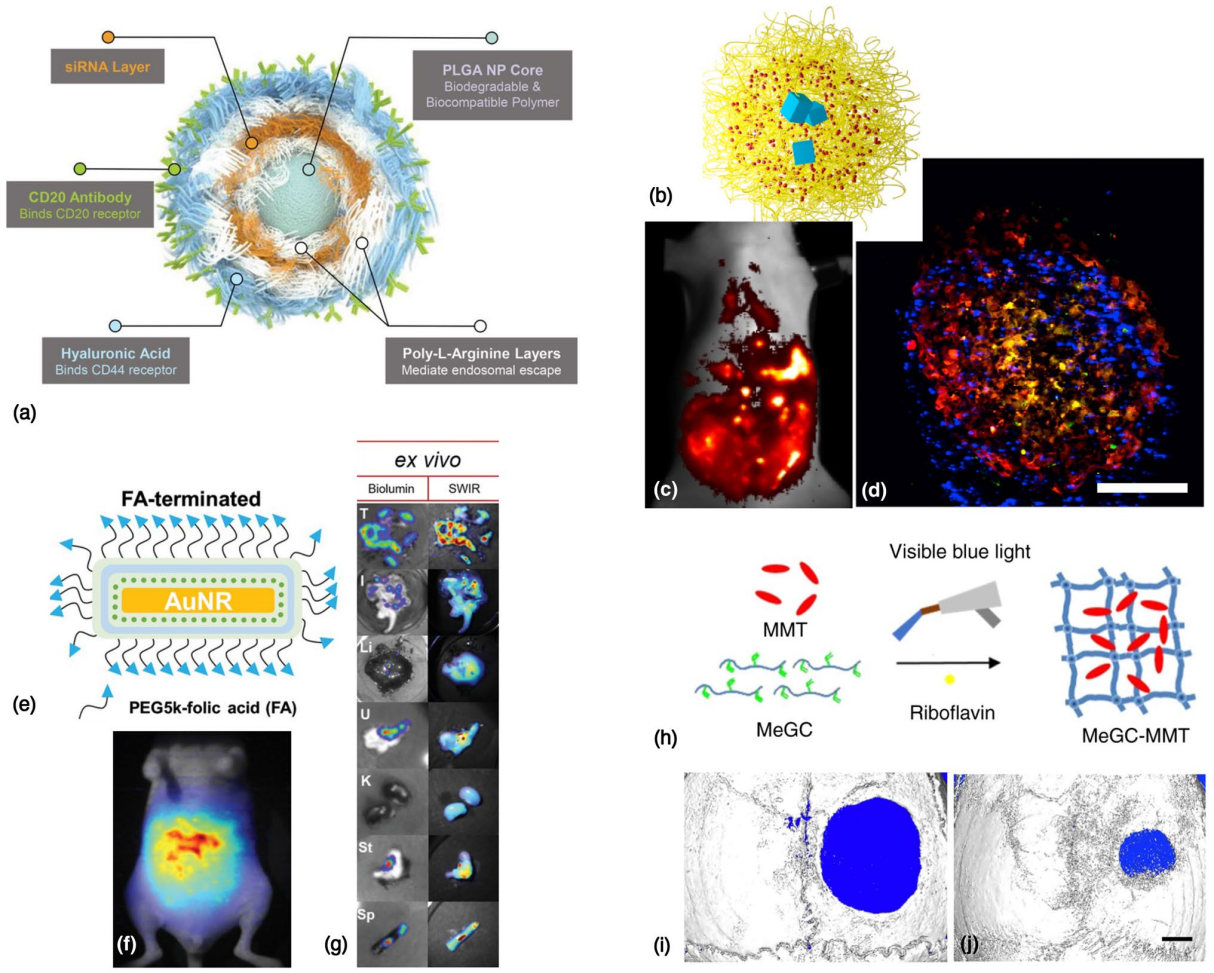
Nanomaterials for cancer therapeutics and tissue engineering: (a) A layer-by-layer (LbL) nanoparticle, around a PLGA core, with dual targeting capability for precision delivery of siRNA therapeutics in difficult-to-target cancers such as blood cancer. Reprinted (adapted) with permission from Choi et al.^[66]^ ©2019 John Wiley and Sons. (b–d) Schematic of (b) PEO-b-PCL nanoparticles (yellow), encapsulating an organic dye, DiR (red) and rare earth lanthanide nanoparticles (LNPs, blue). (c) NIR-II fluorescence of the LNPs revealed the best tumor distribution; confirmed by (d) confocal microscopy, showing co-localization of the LNPs (blue), tumor environment (red) and macrophages (green). Scale bar: $300 \upmu$m. Reprinted (adapted) with permission from Tao et al.^[69]^ ©2017 Elsevier Ltd. (e–g) Plasmon-enhanced SWIR imaging probe: (e) Au nanorods, LbL-coated with polyelectrolyte, and folic acid-targeting; enabling (f) clear tumor distribution visualized in a mouse model of ovarian cancer. (g) Ex vivo harvested organs: strong co-localization with the bioluminescence signal from the tumor cells (T). Reprinted (adapted) with permission from Huang et al.^[70]^ ©2021 John Wiley and Sons. (h) MeGC-MMT nanocomposite hydrogel materials for bone tissue engineering. Comparing in vivo bone regeneration at 6 weeks post surgery in (i) blank (untreated) defects, with very little regeneration $\sim 10\%$, or (j) defects treated with MeGC$+1.5\%$MMT showed $\sim 69\%$ bone growth area. Scale bar: 1 mm. Reprinted (adapted) from Cui et al.^[71]^ CC BY 4.0.

One of the exciting new ways to synthesize new drug delivery systems has been layer-by-layer (LbL) assembly^[65]^: the method of alternating adsorption of two (or more) layers of oppositely charged polyelectrolyte species, with significant advantages in terms of aqueous processing conditions and the ability for high drug loading, along with the capability to coat multiple substrates at various scales ranging from nanoparticles to macroscopic implants. This method also allows incorporation of a variety of therapeutic molecules, such as small molecule drugs, peptides, proteins, growth factors, and nucleic acids, to name a few. As shows in Fig. 5(a), Hammond, P.T. and co-workers have used LbL to formulate a dual-targeted nanoparticle^[66]^ for precision delivery of siRNA. The dual targeting outer layer targets CD20/CD44 positive blood cancer cells, and receptor-mediated endocytosis of the LbL-nanoparticle allows delivery of the siRNA for silencing B-cell lymphoma 2 (BCL-2) mRNA, resulting in significant downregulation of this protein, and helps inhibit the progression of hematologic cancers. Similarly, these nanoparticles have been shown to have applications in a wide range of cancers, such as in improving the efficacy of combination therapies in a glioblastoma model,^[67]^ by leveraging nanoparticles which are able to cross the blood-brain barrier. Furthermore, it is possible to optimize the therapeutic efficacy of LbL NPs by tuning their mechanical properties^[68]^: it was recently discovered that by making changes to the stiffness properties of the liposome core, more compliant LbL nanoparticles offer beneficial trafficking characteristics such as longer elimination half-life, higher accumulation in tumors, and better tumor penetration.

Work done by Bardhan, in collaboration with Belcher et al. has focused on developing nanomaterial-based formulations for the purpose of early detection, diagnostics, and treatment of hard-to-detect cancers, such as ovarian cancer. A palette of different nanomaterials has been tested; each for its unique properties, to achieve complementary applications in imaging of microscopic tumors, with the goal of improved tumor identification for treatment, surgery, or therapeutic intervention. Two such examples are presented in Fig. 5. In Fig. 5(b–d), a core-shell nanoparticle for imaging applications is depicted; composed of diblock PEO-b-PCL copolymers (yellow), encapsulating an organic fluorophore DiR (shown in red), and rare earth lanthanide nanoparticles (shown in blue). As shown in Fig. 5(c), second-window near-infrared (NIR-II) fluorescence from the lanthanide nanoparticles was able to clearly highlight the tumor distribution,^[69]^ in an intraperitoneal mouse model of ovarian cancer, with Fig. 5(d) showing good penetration of the nanoparticles (blue) into the tumor (red). In another example, gold nanorods (AuNR) have been used as a template to decorate short-wave infrared (SWIR) organic dye molecules, with the goal of increasing the fluorescence emission intensity^[70]^ of these dyes. As depicted in Fig. 5(e–g), these nanoparticles were synthesized using the LbL approach, by coating the AuNR-dye structure with a layer of polyethylene glycol (PEG), and using folic acid (FA) as the targeting agent. Using the phenomenon of surface plasmon resonance, the fluorescence emission intensity of the SWIR dyes was increased by a factor of $\sim 45\times$, which makes this AuNR-dye nanoprobe very suitable for use in in vivo imaging applications. In an intraperitoneal mouse model of ovarian cancer, these nanoprobes were used to observe the tumor distribution [Fig. 5(f)], with strong correlation observed ex vivo between the bioluminescence signal from the cancer cells and the SWIR signal from the AuNR-dye in Fig. 5(g). Furthermore, the aforementioned researchers have also designed carbon nanotube-based molecular probes, functionalized with M13 bacteriophage [similar to the construct illustrated in Figure 4(a)], to enable real-time NIR-II fluorescence image-guided surgery in an ovarian cancer mouse model.^[72]^ In the pre-clinical study, compared to the non-guided surgery group (control), the image-guided surgery group showed an improvement in the median survival by over $40\%$, which has the potential to make a meaningful impact in cancer treatment outcomes upon clinical translation, and shows one instance of the power of nanoparticles for cancer theranostics.

Another potential area of interest for the development of nanocomposite materials is the field of tissue engineering and regenerative medicine, where there is significant opportunity for high-performance polymeric materials, hydrogels and nanocomposites,^[73]^ manufactured through additive manufacturing techniques such as 3D printing,^[74]^ to make a meaningful impact. These high-performance polymeric materials have long been sought after in various industries, such as the oil-and-gas industry,^[75]^ for their ability to be rapidly prototyped into functional parts; with the added benefits of excellent chemical resistance, thermal stability and mechanical strength. More recently, 3D-printed hydrogels made of these materials, have enabled “bioprinting”, whereby biomaterials and cells are used to create scaffolds and living tissues.^[76]^ There are numerous considerations to be taken into account to enable successful development of 3D-printed biomaterials: including the printability of the material, its biocompatibility, required mechanical properties, biodegradability, and the ability to mimic natural tissue architecture; each of which are important to be satisfied for biomedically relevant polymer materials, as discussed by Agueda et al.^[77]^ Various nanomaterials are being explored to develop nanocomposites, along with polymer systems, to achieve important functions in biomedical engineering and tissue regeneration through “4D printing” (stimuli-responsive smart materials composed of 3D-printed structures). As an example shown in Fig. 5(d), methacrylated glycol chitosan (MeGC) can be cross-linked, using riboflavin as a photoinitiator, to form a hydrogel network, intercalated by montmorillonite (MMT) clay nanosheets. Using this approach for bone regeneration, it was shown^[71]^ that compared to bone defects left empty after surgery, the MeGC-1.5%MMT nanocomposite showed remarkable bone healing. For a comprehensive overview of the recent progress in the field of 3D printing of polymer nanocomposites, the reader is referred to the excellent review by Advincula et al.^[78]^

Over the years, a number of review articles have summarized the current state-of-the-art in the use of nanomaterial-based platforms for drug delivery applications.^[19, 79–81]^ For an up-to-date summary of the methods to engineer polymeric, lipid-based and inorganic nanoparticles for targeted, precision medicine, the reader is referred to the review by Mitchell et al.^[19]^

## Challenges to applications of nanomaterials in biomedicine: delivery and toxicity issues

While there have been stories of mindblowing successes of LNP-based nanoparticle formulations in the delivery of mRNA-based vaccines for COVID-19 as discussed in Sect. 3, the field has been struggling for quite some time to achieve similar levels of efficacy for tumor treatment. There are two main concerns which need to be accounted for, with the goal of making nanotechnology feasible for clinical translation to help make an impact in effectively managing a broader range of disease conditions.

### Delivery challenges

One of the biggest challenges facing researchers trying to deliver nanoparticles to the site of cancer is the issue of delivering these nanomaterials systemically, and having sufficient amount of these nanoparticles being uptaken at the site of disease for diagnostic or therapeutic application. Since the mid-1980s, the long-standing dogma has been that nano-sized particle formulations, by virtue of their size and long circulation half-life, preferentially traffic to tumors via the enhanced permeability and retention (EPR) effect; allowing for safe and effective drug delivery. However, a growing body of recent evidence from failed clinical trials has begun to challenge this concept,^[82]^ with similar levels of efficacy achieved from the administration of free drug or nano-encapsulated drug. In fact, work done by Chan et al. has questioned the mechanism of targeting of nanoparticles to solid tumors. According to this group, only $\sim 0.7\%$ of the administered dose^[83]^ of the nanoparticle actually makes its way to the site of the disease; which is a rather poor delivery efficiency. More recently, it was shown that the inter-endothelial gaps (up to $\sim 2\upmu$m) in the tumor blood vessels (i.e. leaky vasculature) is responsible for a very small fraction of the nanoparticles transported to the tumor. On the contrary, up to $\sim 97\%$ of the nanoparticles undergo a process of active extravasation^[84]^ through binding and transport via endothelial cells. The knowledge of this delivery mechanism can lead to several new areas of research, in terms of identifying strategies to engineer the tumor endothelium to increase the transport of nanoparticles, and optimize the nanoparticle size, shape and surface chemistry to take advantage of this transport mechanism. Another valid concern to be considered in the design of nanoparticles for systemic delivery is the question of the number of nanoparticles administered (i.e. the number of units of carrier nanoparticle, not the cargo drug or vaccine) - how many is too many? While this is an ongoing area of work, studies in mouse models have shown that a minimum dose threshold for improving tumor delivery is $\sim 1 \times 10^{12}$ nanoparticles.^[85]^ At this suggested dose of a trillion nanoparticles, the dose was high enough to saturate the uptake capacity of Kupffer cells in the liver, thereby leading to enhanced accumulation in the tumor, up to $\sim 12\%$ of the total dose in the tumor. Extrapolating this data on a body weight basis (human liver $\approx 1500\times$ mouse liver), it may be projected that a minimum threshold of $\sim 1.5 \times 10^{15}$ nanoparticles would need to be administered in human patient trials for achieving clinical efficacy.

### Mitigating toxicity concerns

There are various underlying concerns posing the issues of toxicity of nanoscale formulations for biomedical applications. Among some of the major concerns^[86]^ to be addressed are: (i) Genetic toxicity, caused by factors such as DNA damage, mutation, protein adducts, enzyme function dysregulation; (ii) Molecular toxicity, caused by factors such as structural alterations in proteins, conformational changes in protein folding, etc.; (iii) Cellular toxicity, caused by methods such as oxidative stress, generation of reactive oxygen species (ROS), changes in cell motility, cellular binding and persistence, inflammation or tumor angiogenesis; (iv) Tissue toxicity, such as severe inflammation caused by accumulation of nanoparticles in the lungs, or hepatotoxicity caused by positively charged lipid nanoparticles; (v) Physico-chemical toxicity, caused by factors such as the size, shape, surface area, functional groups, mechanism of action of nanoparticles, as well as the route of administration and the physiological microenvironment in which these nanomaterials are delivered.

As a recommendation for the practitioners of nanomedicine, it is strongly encouraged that researchers pay close attention to the aforementioned issues, and take the necessary steps in the early design of their nanomedicine formulations in a “safe-by-design” approach,^[87]^ to avoid ending up with the *aegrescitque medendo* situation (ie. the remedy is worse than the disease). There are many tools available to predict the toxicology of nanomaterial formulations, such as the Quantitative Structure-Activity Relationship (nano-QSAR), Quantitative Nanostructure-Activity Relationship (QNAR), to name a few. The advantage of implementing these *in silico* models is that they combine the knowledge of chemical properties, biology, and the nano-bio interactions at different size scales (from the genomic to tissue-level or whole body) to enhance our understanding of nanomaterial behavior,^[88]^ as well as attempt to provide mechanistic explanation of the predicted or observed nanotoxicity, and can help researchers take steps to mitigate the toxicity concerns before they become roadblocks in the pathway to clinical translation of these novel nanomaterials.

Despite the aforementioned challenges related to delivery efficacy and the concerns regarding the toxicity of nanoparticles, there has been significant progress made, towards the clinical translation and eventual approval of nanoparticle-related formulations of various drugs for diagnostics and therapy. For an up-to-date review of the timeline of such approvals, as depicted in Fig. 1(k), the reader is referred to the excellent, updated series of articles by Anselmo and Mitragotri.^[3–5]^

## Nanotechnology materials in medicine: useful resources for practitioners

We devote a section to listing useful resources for the reader to visit and acquire additional information. Note that although many of these resources are country-specific, there is a wealth of information on these sites to form an informed opinion, and explore further avenues.

- National Nanotechnology Initiative (NNI)﻿ The NNI is an R &D initiative of the U.S. Government. The vision of the NNI is to vision prepare for a future in which the ability to understand and control matter at the nanoscale, leading to technological and industrial revolutions for the benefit of society. The NNI helps interface between $\sim 30$ Federal agencies and departments, to harness knowledge and resources and to facilitate collaborations with academia and the private sector, as appropriate, to promote technology transfer and facilitate commercialization in nanomedicine; among other applications. [US-specific]
- cancer Nanotechnology Laboratory (caNanoLab) This is a data sharing portal, with the goal of facilitating information sharing across the international community working on biomedical nanotechnology , in order to expedite and validate the use of nanotechnology in biomedicine. This lab provides services such as the annotation of nanomaterials with characterizations resulting from physico-chemical, in vitro/in vivo studies, and the ability to share these protocols, data and materials in a standardized, secure manner. [Worldwide]
- ﻿ClinicalTrials.gov This is a database of the U.S. National Library of Medicine at the National Institutes of Health (NIH). This web-based resource maintains a comprehensive database on public- and privately supported clinical studies on a wide array of disease conditions; which is useful to patients and their families, health care professionals, members of the public, as well as researchers and scientists. For example, as of April 2022, the search term “Nano﻿” yields $\sim 410$ results, with $\sim 175$ studies marked as completed, and $\sim 111$ active studies in various stages of recruitment. In another example, applying more specific filters such as “COVID-19” (for the disease condition) and “Nano” as a general search query, there are $\sim 10$ ﻿studies, of which 2 are completed, 5 are recruiting, and the remaining are not recruiting. This is also a valuable resource for researchers to explore prior to starting a new research project, as it helps avoid duplication of effort if there are already similar nanomaterials in an advanced stage of development with an ongoing or proposed clinical trial. [U.S. and Worldwide]
- ﻿U.S. FDA - Guidance for Industry This document, “Drug Products, Including Biological Products, that Contain Nanomaterials—Guidance for Industry”, is the final guidance document published by the FDA and related government agencies (U.S. Department of Health and Human Services (HHS), Center for Drug Evaluation and Research (CDER), and Center for Biologics Evaluation and Research (CBER)) in April 2022. It includes useful guidance regarding quality recommendations for nanomaterial products, non-clinical studies, as well as considerations for clinical development, including the 505(b)(2), 505(j) and 351(k) regulatory pathways for new drug development. [US-specific]
- nanoHUB This is a free, open cyber-infrastructure platform to enable and support computational research and collaboration in nanomaterials under the auspices of the Network for Computational Nanotechnology (NCN), funded by the U.S. National Science Foundation (NSF); established with the goal of supporting the NNI. As of 2020, this resource boasts $\sim 600$ simulation codes or apps, $> 100$ courses, and other useful information such as links to scientific papers in the field of nanotechnology. [Worldwide]
- ﻿National Center for Nanoscience and Technology (NCNST) This is an outstanding center of excellence, co-founded by the Chinese Academy of Sciences (CAS) and the Ministry of Education of the People’s Republic of China. It has 3 nano-focused CAS Key Laboratories: The Lab for Nanomaterials & Nanosafety, Lab for Standardization and Measurement for Nanotechnology, and the Lab for Nanosystem and Hierarchical Fabrication. The NCNST is also world-renowned for collaborative science and technology exchange with international partners such as the US, Canada, UK, Japan, Germany, South Korea, etc., through joint projects, fellowships and other training opportunities, as well as multi-lateral international conferences. [China-specific]
- ﻿Mission on Nano Science and Technology (Nano Mission) The Nano Mission is an umbrella program for the development of research in the growing field of nanoscience and technology, funded by the Department of Science and Technology of the Government of India. It also funds research proposals for scientists and faculty at all eligible institutions in India. [India-specific]
- ﻿DaNa 4.0 Sponsored by the German Federal Ministry of Education and Research, DaNa4.0 is focused on studying new, advanced materials including nanomaterials, whether they can be harmful to humans, animals and the environment. This initiative also creates a knowledge base by analyzing scientific literature on the human and environmental toxicology of new, advanced nanomaterials. [Germany/Swiss-specific]
- ﻿WHO Nanotechnology and human health Resources from an expert meeting convened by the World Health Organization to develop and use improved assessment methods for evaluating the health risks as well as the benefits of using nanomaterials. This report called for a “risk governance” model to study the connections between nanotechnology and human health. [Worldwide]
- ﻿Nanowerk Catalog This is a free directory of nanomaterial suppliers and vendors.

## Outlook and future prospects

**Figure 6:**
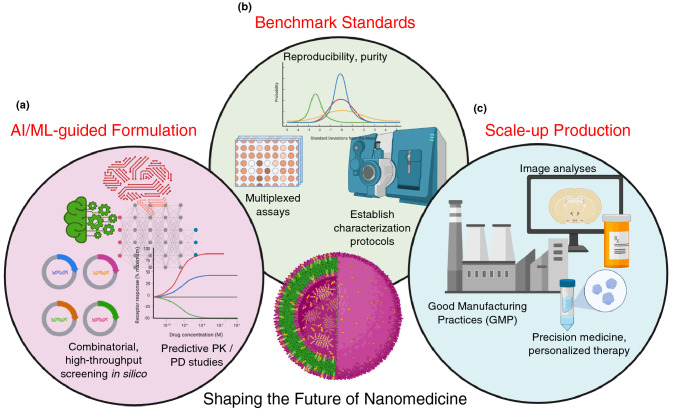
Focus areas for the rapid development of Nanomedicine. (a) Expanding the role of AI/ML-based techniques in nanomaterial formulation; (b) establishing a set of industry standards and robust protocols to ensure purity and reproducibility; and (c) Achieving processes to scale-up nanomedicine formulations in accordance with regulatory practices to enable targeted, precision medicine for personalized therapy. Created with Biorender.com

Figure 6 summarizes the key focus areas of importance to be addressed for strengthening the field of nanomedicine, each of which we elaborate below.

### Machine learning in nanomaterials for biomedicine

Till date, the field of nanomedicine has been mostly dominated by designing nanomaterials on a case-by-case basis; tailored to a very specific application (one formulation, screened against one cell line or disease model of interest).^[89]^ As a result of this design philosophy, there has been very limited integration of the tools required for biological screening of these nanomaterials. While the field of drug development has certainly benefit from the myriad computational and simulation tools available through the rise of artificial intelligence (AI) and machine learning (ML) techniques developed over the last decade,^[90]^ the same, unfortunately, hasn’t been realized to a significant extent in the field of nanomedicine. One of the limiting factors in the development of AI/ML tools for nanotherapeutics is that because of their highly specific targeted nature and the (relatively) few cases of FDA-approved products already in the clinic, there is a shortage of large datasets of well-compiled, annotated data for researchers to mine through and build AI models upon. Despite these limitations, researchers are coming up with creative ways to use ML for predictive design of nanomaterials. For example, Mirkin et al. have managed to synthesize 18 new complex heterojunction nanomaterials^[91]^ using data from an 8-dimensional chemical space (consisting of Au, Ag, Cu, Co, Ni, Pd, Sn and Pt as inputs) using ML-assisted optimization. Along these lines, there has been a recent push for high-throughput screening and machine learning to explore the design space for nanomaterials useful in biomedical applications. In one instance, Yamankurt et al.^[92]^ have screened for the activity-structure relations of spherical nucleic acid nanoparticle libraries as candidates for cancer vaccines. Coupled with a high-throughput method to synthesize these candidate nanoparticles, the authors tested the ability of the products to elicit an immune response by activation of the TLR9 signaling pathway. In more recent work, Hammond et al. have reported the first high-throughput combinatorial screen of nanoparticles with cancer cell lines,^[93]^ through a pooled screening technique, and used this to identify predictive biomarkers for the interaction of nanoparticles with cancer cells. While these recent examples provide encouraging demonstration of the power of ML-based toolkits applied to nanomedicine, a word of caution is in order: many of the ML-based solutions used in other fields promise to offer “the best output” (whether it be a new candidate compound, or the results of an image processing pipeline) with no information about the physical interpretation of the output solution (essentially a “black box” approach). However, this approach would be dangerous to apply in the healthcare sector, and it is because of this reason that AI/ML alone is of limited utility. There is a strong need to (a) combine the power of AI-based techniques with the mechanistic insights derived from modeling/simulation of the system^[94]^ under study; and (b) test the validity of the AI/ML model’s output with real experimental data, wherever possible, before generating conclusions about the usefulness of these approaches. For example, Advincula, R. and co-workers have demonstrated a technique of combining ML with finite element analysis and empirical experimental data to obtain insights about the precise mechanical properties at the interface^[95]^ of composite materials; an approach which can be suitably adapted to study the interface of bio-nano materials.

As a recommendation for researchers working towards next-generation nanomedicine formulations, it is strongly desirable to have a centralized, annotated database of a library of nanomaterial compounds, their formulations, physico-chemical characteristics, and most importantly—the bio-nano interactions with different types of cell lines or tissues. It is worth looking at other related fields of medicine for inspiration on how to design a valuable, useful database. For example, the publicly available ﻿The Cancer Genome Atlas (TCGA) under the purview of the NCI and the National Human Genome Research Institute of the U.S. NIH, has been a deep source of multi-omics knowledge for making important discoveries^[96]^ about the fundamental biology playing a role in 33 types of cancer. However, in order to achieve this high level of data quality for nanomedicine, it is of utmost importance to have sets of standardized assays and characterization protocols to ensure data quality, integrity, and reproducibility across various research labs. This is discussed in the next section.

### Standardization of tests and assays for characterization of new nanomaterials

One of the key issues that need to be urgently addressed, in order to enable new nanomaterials to make the jump from bench-to-bedside, is the development of strong, robust and reproducible standard tests and assays for characterization of nanomaterials.^[97]^ According to the U.S. FDA, drug products that include nanomaterials in their formulation are classified as “non-biological complex drugs” (NBCDs). These are a class of medical products which are distinct from conventional small-molecule drugs, and are made from different components which result in a hybrid structure that challenge the existing techniques used in standard pharmaceutical analyses. Due to the complex nature of NBCDs, even small variations in the manusfacturing process can result in significant differences in the composition, efficacy, safety and toxicologic profile of these nanomedicines. To keep up with the explosion in the rapid development of new, next-generation, “smart” nanomedicine formulations, there is an urgency to develop regulatory guidelines for the evaluation of these nanomaterials; with the hope of clinical deployment in a rapid yet safe manner. There are several federal agencies working on these issues; such as the project “Metrology for Nanomaterials in Medicine” funded by the National Institute of Standards and Technology (NIST) of the U.S. Department of Commerce. For example, researchers from the NIST group have compiled a list of the available state-of-the-art technologies for performing particle size and distribution analysis^[98]^ for novel, complex nanoparticle formulations. For a perspective of the regulatory issues in the context of the European Union, the reader is referred to the excellent review by Soares et al.^[99]^

As listed on the *Good*Nano*Guide*, a number of nanotechnology standards already exist, or are in various stages of development, by standard developing organizations such as the ASTM International, ISO, IEEE, BSI and others. The reader is referred to the standards set by ASTM International: Subcommittee E56.08 on Nano-Enabled Medical Products, and the ISO/TC 229 Nanotechnologies technical committee. For the latter, as of April 2022, there are 98 published ISO standards, and 29 standards under development. Researchers also need to make a concerted effort to study the nano-bio interface,^[100]^ and not just evaluate the properties of their as-synthesized nanomaterial in isolation, as these interactions of nanomedicine with biological systems will play a strong role towards the successful deployment of these nanomaterials in clinical applications. As a recommendation for good practice, to be used as a guideline while designing and reporting data on new nanomedicine products, researchers are suggested to use tools such as the “Minimum Information Reporting in Bio-Nano Experimental Literature” (MIRIBEL)^[101]^ standard checklist; which is guided by the principles of Reusability, Quantification, Practicality and Quality of the nanomaterial data.

### Clinical translation: removing the barriers to product launch

Finally, in the last but arguably the most important step in the nanomedicine development cycle, there is the need to develop a robust pipeline to achieve scalable, controlled and reproducible manufacturing of nanomedicines^[102]^ in accordance with current good manufacturing practice (CGMP) regulations, including fundamental understanding of the property characterization, clinical and regulatory issues^[103]^ to enable the translation of these new therapeutics from the bench-to-bedside. There are several barriers which need to be overcome, during the development cycle of a new nanomedicine product: (a) Physico-chemical characterization; (b) Biocompatibility and Toxicology studies; (c) Pharmacokinetics and pharmacodynamics assays; (d) Process control; (e) Scale-up and reproducibility.

One can draw several important lessons from the rapid, unprecedented scale-up of the mRNA COVID-19 vaccines used to inoculate a large fraction of the world’s population. The large-scale production of these vaccines entails two main steps^[104]^: (i) Upstream processing, which consists of a cell-free process to produce the in vitro-transcribed mRNA followed by capping; and (ii) Downstream processing, which consists of a series of steps (such as DNAse digestion, LiCl precipitation, chromatography, etc.) to purify the mRNA product, complemented with the formulation of the LNP delivery system, and Fill-to-Finish steps. While there are several techniques now available for achieving high-purity synthesis of a new product formulation at the lab scale, the challenge is to make these techniques work when the process needs to be scaled up^[105]^ to millions, or even billions of doses (as a vaccine candidate) to ensure batch-to-batch consistency.^[106]^ Given the enormous complexity, cost and time-consuming nature of translating a novel nanomaterial therapeutic from the lab to the clinic, a recommendation for good practice is in order: it is important to perform a cost-benefit analysis,^[107]^ with a clear indication of a positive, worthwhile benefit-to-risk ratio, prior to starting lab-scale (pre-clinical) development of any new nanomaterial formulation. The cost-benefit can be obtained in several ways: (a) as an overall reduction in health care costs by increasing therapeutic efficacy through a tailored “personalized medicine” approach (thus requiring lower dosage of nanomedicines, compared to conventional therapies); (b) enhanced quality of life for patients, by reducing adverse events or instances of systemic/off-target toxicitiy; (c) reducing the need for surgery or other high-risk invasive interventions, and so on. It is only by presenting a clear business case that the pharmaceutical industry will be convinced to budget their R &D dollars for enabling the clinical translation of these novel nanomedicines.

Finally, just as materials researchers hope to learn and apply the lessons learned from the rapid development of COVID-19 vaccines to the development of therapeutic and diagnostic solutions for cancer and other disease conditions, there is a parallel community of scientists looking to investigate the knowledge learned from the field of nanomedicine to understanding the causes of rare adverse reactions^[108]^ to the LNP-based COVID-19 vaccines. It is this wonderful iterative process of learning and self-reflection which ultimately leads to more informed decision-making about the design principles of nanomedicine formulations; thereby leading to better safety, efficacy and tolerability profiles for all individuals.

## Data Availability

Not applicable
